# A preliminary analysis of the effect of individual differences on cognitive performance in young companion dogs

**DOI:** 10.1007/s10071-024-01868-4

**Published:** 2024-04-01

**Authors:** Jordan G. Smith, Sarah Krichbaum, Lane Montgomery, Emma Cox, Jeffrey S. Katz

**Affiliations:** 1 Department of Psychological Sciences, Auburn University, Auburn, AL USA; 2 Canine Performance Sciences, College of Veterinary Medicine, Auburn University, Auburn, AL USA; 3 Auburn University, 104 Greene Hall, Auburn, AL 36849 USA

**Keywords:** Individual differences, Cognition, Development, Puppies, Executive function

## Abstract

Many factors influence cognitive performance in dogs, including breed, temperament, rearing history, and training. Studies in working dog populations have demonstrated age-related improvements in cognitive task performance across the first years of development. However, the effect of certain factors, such as age, sex, and temperament, on cognitive performance in puppies has yet to be evaluated in a more diverse population of companion dogs. In this study, companion dogs under 12 months of age were tested once on two tasks purported to measure aspects of executive function: the delayed-search task (DST) and the detour reversal task (DRT). Owners also filled out the Canine Behavioral Assessment and Research Questionnaire (C-BARQ) to evaluate how temperament influenced task performance. Contrary to prior research, performance did not improve with age on either task. However, the lack of age effects was likely the result of small sample sizes and individual differences across other factors influencing performance. Specifically, temperament differences as measured by the C-BARQ subscales for nonsocial fear and excitability predicted task performance on the DST, but the effect of temperament on task performance differed between males and females. Excitability also predicted performance on the DRT, but the effect depended on the age of the dog. In addition, no correlations were observed between task measures, indicating a lack of construct validity. Overall, these findings provide a preliminary analysis of factors that appear to influence cognitive task performance in young companion dogs and highlight suggestions for future research evaluating the impact of individual differences on cognitive performance.

**Supplementary Information:**

The online version contains supplementary material available at 10.1007/s10071-024-01868-4.

It is generally understood that a multitude of factors can affect cognitive task performance when comparing across species (Auersperg et al. 2011; Cleal et al. 2021; Guillette et al. 2017; MacLean et al. 2014, 2017; Marshall-Pescini et al. 2015, 2017; Rowe and Healy 2014). However, research has also demonstrated that specific factors can affect cognitive task performance when comparing individuals within species as well (e.g., Arden et al. 2016; Hare et al. 2005). In this study, we evaluated how individual differences in young companion dogs can influence cognitive task performance.

The domestic dog (*Canis familiaris*) is a species known for its diversity, both behaviorally and morphologically (Ruple et al. 2022). While much of this diversity is driven by differences between breeds (Hart and Hart 2016), variation between individuals is also a factor of the number of different environments dogs experience (Foraita et al. 2021a). In relation to cognition, this variation between individual dogs can lead to many different factors affecting performance on tasks measuring cognitive and problem-solving abilities. Factors such as training (Barrera et al. 2019; D’Aniello et al., 2015; Foraita et al. 2023; Lazarowski et al. 2020; Marshall-Pescini et al. 2008, 2009; Scandurra et al. 2015), rearing history (Duranton and Gaunet 2016; Lazarowski and Dorman 2015; Fagnani et al. 2016), temperament (Bray et al. 2015; Bray, Sammel, Seyfarth, Bray et al. 2017a, b; Marshall-Pescini et al. 2008), and breed (Gnanadesikan et al. 2020; Passalacqua et al. 2011) have all been shown to influence cognitive and problem-solving abilities.

While differences in cognitive performance on specific tasks could be due to non-cognitive factors such as context (Bray et al. 2014), motivational differences (Marshall-Pescini et al. 2017), or learning history (Duranton and Gaunet 2016), it appears that specific early life experiences may have a more direct influence on the development of executive function in dogs. Executive function (EF) is a combination of abilities that allow organisms to respond appropriately given the context and adapt to changes in their environment (Olsen 2018). EF is often categorized into several different components: working memory, inhibitory control (often divided into behavioral inhibition and cognitive inhibition/attention), and flexibility (Diamond 2013; Foraita et al. 2021a). Foraita et al. (2021a) discusses several different environmental factors that appear to influence the development of EF in dogs, including maternal care (Bray et al. 2017), housing (Lazarowski and Dorman 2015), and training (Barrera et al. 2019). The authors posit that stress could be functioning as a mediating factor, with stressful experiences negatively impacting the development of EF in dogs, although more research is needed to evaluate this relationship (Foraita et al. 2021a). Ultimately, individual differences in EF should be taken into consideration when assessing cognitive performance, as baseline differences in EF, if not properly controlled for, could influence research findings attempting to evaluate the impact of other variables on cognitive task performance. This factor is specifically relevant when testing a companion dog population; namely, companion dogs are raised in highly variable environments which could differentially impact the development of EF, and consequently, cognitive task performance.

Age also affects cognitive performance in dogs, with most dogs displaying a quadratic trajectory of cognitive aging marked by a period of development early in life and a decline in these same abilities later in life (Watowich et al. 2020; although see Foraita et al. (2023) for more complex associations with age for certain cognitive abilities). Because models of cognitive aging in dogs have translational value to neurodegenerative diseases in humans, many studies have evaluated cognitive performance in older dogs, noting a general decline in cognitive abilities with age (Bray et al. 2014; Tapp et al. 2003; Wallis et al. 2016). However, others have found that the rate of cognitive decline not only varies between individuals (Adams et al. 2000) but may also be directly influenced by certain life experiences (e.g., physical exercise, social engagement, training experiences) (Bray et al. 2022; Chapagain et al. 2018; Yarborough et al. 2022). Although much is known about cognitive decline in the latter part of a dog’s life, only a few studies have evaluated the ontogenetic development of cognitive abilities in dogs during the first years of life. While evidence of executive function is present as early as 7.5-8 weeks of age (Bray et al. 2020; Foraita et al. 2021b), performance on cognitive tasks does increase over the first 1–2 years of life (Bray et al. 2021; Lazarowski et al. 2020b). Specifically, Bray et al. (2021) observed age-related improvements on measures of inhibitory control and flexibility. Lazarowski et al. (2020) observed similar findings to Bray et al. (2021) as well as developmental increases on a measure of attention and working memory. Differences in cognitive abilities also appear to be evident at an early age (Foraita et al. 2021b), with some differences remaining stable across development (Bray et al. 2021; Lazarowski et al. 2020b). Although these studies provide a foundational understanding of the ontogenetic development of cognitive abilities in dogs, these reports have focused on relatively homogenous working dog populations which often experience very similar early life experiences. Therefore, considering variations in early life experiences could influence the development of EF in dogs, it is important to understand both the impact of age and other individual differences on cognitive task performance in a young and potentially diverse population of dogs.

We selected two tasks purported to measure aspects of EF to evaluate the factors impacting cognitive performance in a population of companion dogs under 12 months of age. Specifically, we tested puppies on a measure of inhibitory control and flexibility (i.e., detour reversal task; DRT) and on a measure of attention and working memory (i.e., delayed search task, DST). The DRT, also known as the A-not-B barrier task, has been used as a measure of inhibitory control in dogs (Lazarowski et al. 2020) and requires a dog to learn how to navigate around one side of a barrier during an acquisition phase, followed by a reversal phase in which the dog must inhibit responding to the previously rewarded side and instead navigate around the other side of the barrier (Osthaus et al. 2010). Reversal tasks are also used to measure flexibility because participants must shift responding in one direction in favor of responding in the opposite direction (Olsen 2018). The DST (other names include the visible displacement task, the delayed response task, and the object choice task) has previously been used as a measure of spatial working memory in dogs (Bray et al. 2021; Foraita et al. 2021b; Krichbaum et al. 2021). In this task, a reward is hidden in one of two or three locations, and the dog is released to make a choice after a specified delay. While some studies with dogs have attempted to control for other strategies apart from working memory that may be used during this task (i.e., placing a screen between the subject and the reward locations during the delay to prevent fixation on the location of the reward as in Foraita et al. (2021b)), other studies posit that without controlling for cues related to body orientation, this task may allow dogs to use aspects of attention in addition to working memory (Krichbaum et al. 2021; Lazarowski et al. 2020b). Krichbaum et al. (2021) demonstrated that even with a screen placed in front of the reward locations during the delays, dogs that oriented their head or body in the direction of the correct location for a greater percentage of the delay time also achieved a higher percentage of correct trials. However, dogs never oriented towards the reward location for the entire delay duration, indicating performance on this task may rely on a combination of working memory and attention. Because we did not control for fixation on the reward location or body orientation during the DST in this study, performance on this task likely reflects aspects of both working memory and attention.

Given the factors that influence cognitive performance in dogs, we hypothesized that differences in age and temperament would influence performance on both tasks. Specifically, we predicted that older puppies would perform better than younger puppies on both tasks based on the findings of Lazarowski et al. (2020) demonstrating that performance on these tasks increased as a function of age in candidate detection dogs. In regard to temperament, we asked the owners to complete the Canine Behavioral Assessment and Research Questionnaire (C-BARQ), a validated survey designed to evaluate temperament in companion dogs (Hsu and Serpell 2003). Because the tasks used in this study supposedly measure aspects of EF, we predicted that nonsocial aspects of dog temperament would most significantly impact performance on these tasks. Specifically, we were interested in scores on the C-BARQ subscales for trainability, excitability, and nonsocial fear. Previously, trainability has been positively associated with problem-solving abilities (Bray et al. 2017b; Marshall-Pescini et al. 2008). In contrast, fear of nonsocial stimuli negatively impacts problem-solving abilities (Overall et al. 2019); however, other studies found that nonsocial fear did not influence performance on a problem-solving task (Bray et al. 2017b; Marshall-Pescini et al. 2008). Excitability also appears to negatively influence cognitive task performance, with greater excitability leading to impaired performance on a memory problem-solving task and a sustained attention task (Bray et al. 2017). The effect of excitability is also specifically influenced by the level of arousal the dog experiences during testing, leading to worse performance on an inhibitory control task in excitable dogs (Bray et al. 2015). Therefore, we hypothesized that individual differences in temperament as assessed by the C-BARQ would predict performance on both tasks. Specifically, trainability would positively predict performance on the DST and DRT whereas excitability and nonsocial fear would predict a negative relationship. Lastly, we predicted that correlations between tasks would demonstrate construct validity of the two tasks as measures of EF, such that an increase in performance on the DST would be associated with an increase in performance on the DRT.

## Methods

### Subjects

For this study, companion dogs from 3 to 11 months of age were recruited from the local community. Participants were recruited through several methods, including the Auburn University School of Veterinary Medicine email listserv, Facebook ads targeted to the local area, and through client connections at a local dog training facility. Overall, 48 puppies (21 M; Age (in months): mean [*M*] = 6.44, standard error [*SE*] = 0.34, range = 3–11) were successfully recruited for the study. Breed, neuter status, and information on where the dog was obtained (e.g., rescue, stray, or breeder) were collected for each dog (Table S1). Ethical approval for this research was granted by the Auburn University Institutional Animal Care and Use Committee (Protocol # 2020–3725).

### General testing procedures

Testing occurred at two different locations over the course of the study due to changes in accessibility of certain locations as a result of COVID-19. The first location was a local dog training facility (ReKalibrated K9) that conducts weekly puppy classes as well as one-on-one training sessions. A testing arena (3 × 3 m) was set up in the central training room using either a canopy tent with walls or plastic lattice gates. The other location was a behavioral research laboratory (3.4 × 5.5 m) at the Auburn University MRI Research Center used for various dog projects.

Prior to testing, the owner was briefly familiarized with the methods associated with both tasks and was given a consent form to sign. All puppies were handled by an experimenter for both tasks, but the owner remained in the room for testing to avoid issues related to separation anxiety. Owners were given paperwork to complete and were instructed to avoid interacting with their puppy throughout testing. Owners were also asked whether their puppy preferred a food reward or a toy reward to ensure the puppy’s preferred method of reinforcement was used during testing. Food rewards were Bil-Jac Treats®, Pet Botanics® Training Rewards^TM/MC^, and Charlee Bear Treats®. Toy rewards were medium and large Chuckit!® balls and a small Kong® Wubba.

Each puppy was tested on the DST and the DRT, with task order counterbalanced across puppies. Testing lasted approximately 30 min for each puppy and participants were only tested once. All testing was video recorded using a GoPro® Hero 8. Choice was live-scored for both tasks, but latency to cross the barrier on the DRT was scored after testing using the video recording.

### Delayed-search task procedures

**Fig. 1:**
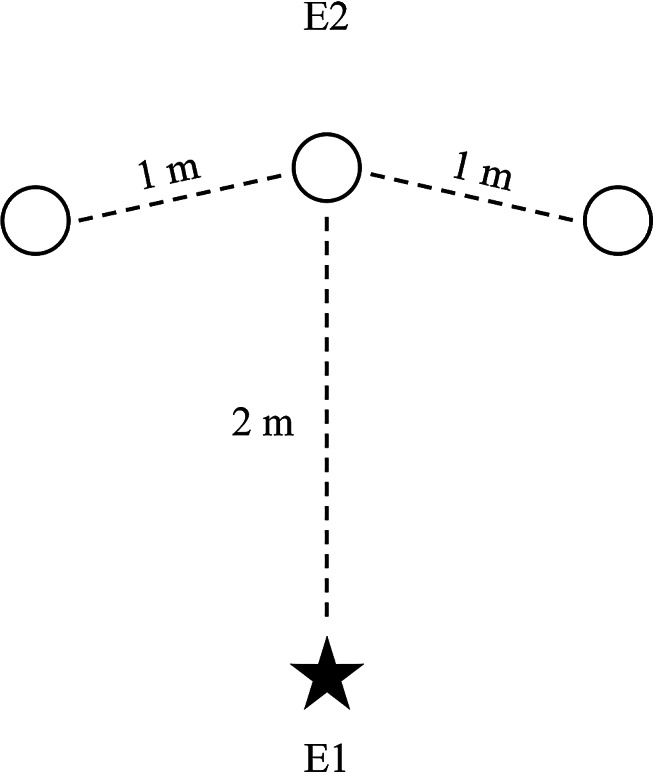
Illustration of the test set-up for the DST (not drawn to scale). Experimenter 1 (E1) held the puppy (represented by the star) at the starting location. Experimenter 2 (E2) placed the reward in one of the three buckets (represented by the circles) and then returned to the starting position behind the middle location facing away from the puppy

For the DST, three different hiding locations were used to measure the effect of delay on object-search abilities similar to methods used by Lazarowski et al. (2020). Three buckets (small: 12.7 × 14.6 cm; large: 18.4 × 20.3 cm), open-end up, were each placed 1 m apart from each other and 2 m away from the starting location. The size of the buckets used depended on the size of the puppy at testing to prevent larger puppies from seeing inside the smaller buckets, but the buckets were visually similar in all other aspects. Experimenter 1 (E1) held the puppy at the starting location while Experimenter 2 (E2) stood behind the middle bucket facing away from the puppy (Fig. 1).

The task began with a series of warm-up trials in which a 0-second delay occurred between the placement of the reward and the puppy’s release to make a choice. These trials were conducted to ensure the puppy could reach a level of performance that was significantly above chance with a 0-second delay before testing the puppy on longer delays. For these trials, E2 called out to the puppy and showed it the reward to get its attention and then placed the reward in one randomly determined bucket. After placing the reward in the bucket, E2 returned to the middle location facing away from the puppy. Once E2 was in position, E1 released the puppy to make a choice. In this task, choice was defined as the first bucket the puppy’s snout came within 10 cm of the top of the bucket after being released. If the puppy chose the bucket with the reward, it was allowed to retrieve the reward from the bucket and the trial was marked as correct. If the puppy chose either of the two empty buckets or did not make a choice within 30 s of being released, the trial was marked as incorrect and the next trial began. Puppies were permitted to search and retrieve the reward from the correct bucket on all trials even if their initial choice was incorrect to maintain motivation throughout testing. If the puppy chose correctly for 5/6 consecutive trials, then delay testing proceeded. If the puppy did not meet the warm-up criteria within 12 trials, the task ended.

The delay portion of the task consisted of nine total trials at three different delays: 0, 10, and 20 s. Delays were based on other studies that tested puppies on this task (Bray et al. 2021). The trials were balanced for delay (i.e., 3 trials at each delay) and location, but the order of trials was pseudorandomized across sessions, with the same delay and location occurring no more than two times in a row. Trials with a 0 s delay followed the same procedure used for the warm-up trials. For trials with a delay of 10–20 s, the delay started when E2 returned to the starting position, and ended when E2 said “okay” which communicated that the trial could begin. At this point, E1 released the puppy to make a choice. If a puppy did not make a choice within 30 s of being released, the reward was removed from the correct bucket by E2, and the next trial began. However, to avoid confounding effects related to motivation during the delay portion of the task, dogs that did not make a choice on a trial during testing with delays were removed from analyses. The dependent measure for this task was the percentage of correct trials at each delay.

An odor control procedure was also implemented for this task to ensure that puppies were not using odor cues to find the reward. Puppies that passed the warm-up criteria and got more than 3 trials correct during the delay portion of the task were tested on the odor control procedure. This procedure consisted of 6 trials in which the puppy was removed from the room or turned away from the testing arena while E2 placed the reward in 1 of the 3 locations. Puppies that performed significantly above chance on the odor control trials (i.e., 5/6 trials correct) were automatically removed from analyses.

### Detour reversal task procedures

**Fig. 2:**
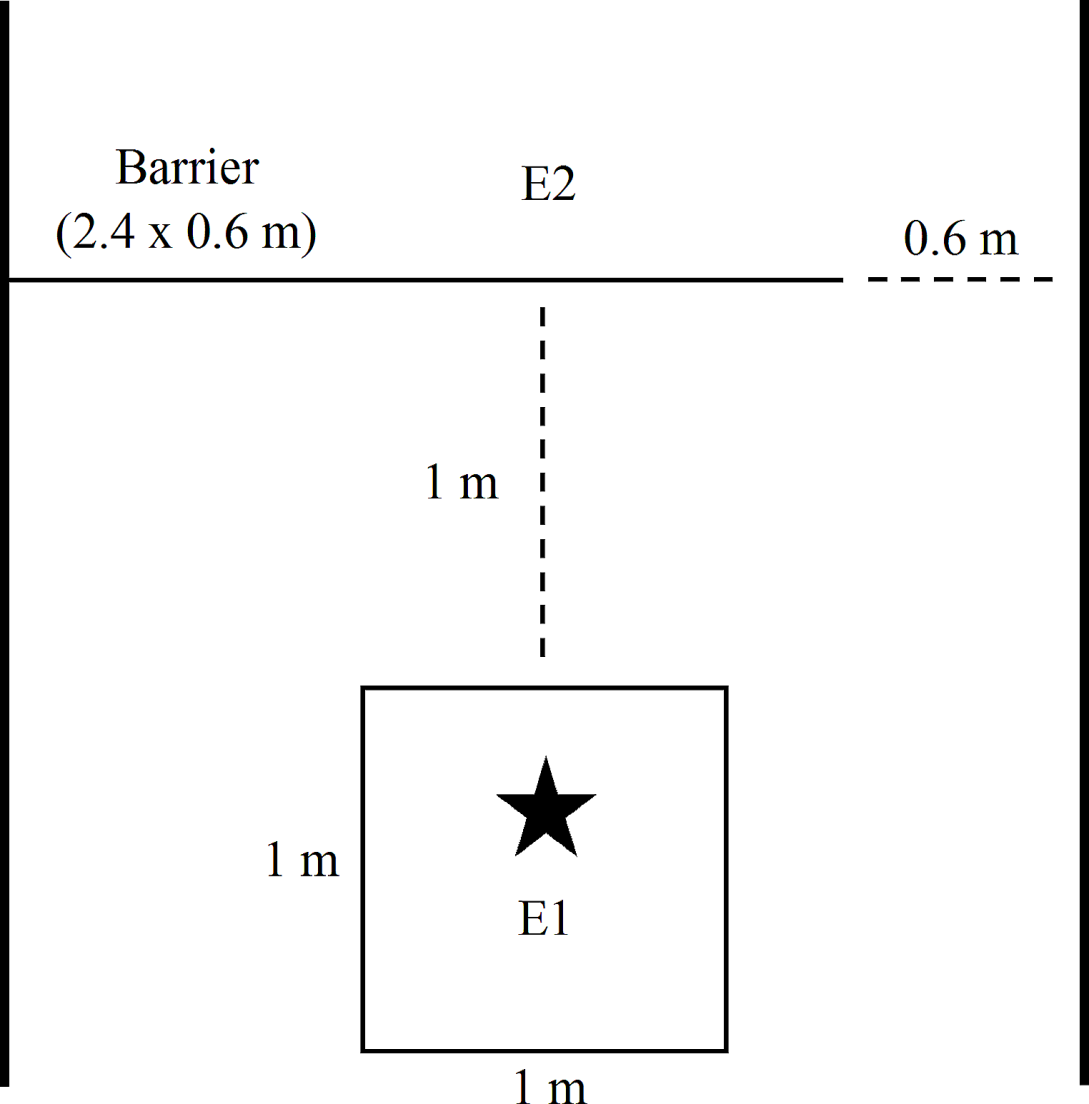
Illustration of the test set-up for the DRT (not drawn to scale). Experimenter 1 (E1) held the puppy (represented by the star) at the starting location, a 1 × 1 m square marked by tape on the ground. A barrier separated the puppy from Experimenter 2 (E2) with the reward so that the puppy had to navigate around the barrier through an opening on one side of the room. A correct choice was defined as the puppy stepping out of the starting location with at least one front paw on the side corresponding to the barrier opening. During the reversal phase of the task, the barrier was moved so that the opening was switched to the opposite side of the room

The DRT was based on methods used by Lazarowski et al. (2020) and Osthaus et al. (2010). For the DRT, a movable gate (2.4 × 0.6 m) made of plastic lattice attached to PVC was used to create a transparent barrier between the puppy and E2. The gate was pushed up against one side of the testing area, resulting in a 0.6 m gap between the gate and the wall on the other side of the testing area. The puppy was held by E1 at the starting location, a 1 × 1 m square marked on the ground with tape, facing the barrier. The starting location was located 1 m away from the barrier and was positioned at the midpoint of the width of the testing arena so that the puppy was equidistant from both walls on either side of the testing arena. E2 was positioned at the midpoint of the testing arena on the other side of the barrier facing the puppy and E1 (Fig. 2).

The first trial of the acquisition phase began when E2 called the puppy’s name, which acted as a signal for E1 to release the puppy from the starting location. The puppy was given 30 s to navigate around the barrier, and if 30 s passed without the puppy crossing the opening of the barrier, the puppy was guided to the opening of the barrier by E1 and E2. After crossing the barrier opening, the puppy was reinforced with its preferred reward (as reported by the owner) by E2 and escorted back to the starting box by E1. In this task, choice was defined as the first side of the starting location the puppy stepped over with at least one front paw after being released, with a correct choice involving the puppy stepping over the side of the starting location associated with the opening of the barrier (e.g., if the barrier opening was to the left of the puppy from the starting location, a correct choice would involve the puppy stepping over the left side of the starting location after being released). The puppy could approach the barrier after being released and still make a correct choice as long as it crossed the correct side of the starting location prior to crossing the incorrect side. This process was repeated three more times for a total of four acquisition trials at which point the puppy was removed from the testing arena while the barrier opening was switched to the opposite side of the room for the reversal phase. The puppy was then brought back into the testing arena to complete four reversal trials following the same methods used in the acquisition trials. The order of the side of the barrier opening was counterbalanced across puppies.

The number of correct trials across the acquisition and reversal phases were collected along with the trial number of the first correct reversal trial. Latency to cross the barrier opening on the last acquisition trial and the first reversal trial were also collected to analyze the difference between the two latencies (referred to as the difference score), with a larger difference score indicating that the puppy required more time to navigate around the barrier on the first reversal trial relative to the last acquisition trial. If the puppy was unable to cross the barrier opening within 30 s, a latency of 30 s was scored for that trial.

## C-BARQ

The C-BARQ (Hsu and Serpell 2003) was given to each owner to fill out while their puppy completed both tasks. Subscales for Trainability, Excitability, and Nonsocial Fear were calculated by averaging the scores from all items pertaining to the subscales. For the Trainability subscale, items 1 through 8 were averaged after items 5 through 7 were reverse scored. Items 38, 41, 42, 44, 47, and 48 were averaged to calculate the Nonsocial Fear subscale, and items 63–68 were averaged to calculate the Excitability subscale. Items for each of the subscales are listed in Table S2.

### Statistical analyses

For the DST, a generalized linear mixed-effects model (GLMM) was conducted with percent correct [continuous variable] at each delay on the DST as the dependent variable and delay [continuous variable] as a fixed factor with subject ID as a random factor. One-sample *t*-tests were also conducted to compare percent correct at each individual delay (0, 10, and 20 s) to chance (33%). These models were conducted to confirm that a typical delay function was observed for the DST and that performance was significantly above chance at each delay prior to conducting further analyses in which percent correct was collapsed across delay.

To measure the effect of age and temperament on our dependent measures, we conducted generalized linear models (GLMs) for overall percent correct on the DST and the dependent measures associated with the DRT (i.e., total number of correct reversal trials [count variable], trial number of the first correct reversal [count variable] and the difference score [latency (s) on the first reversal trial – latency (s) on the last acquisition trial; continuous variable]). To reduce the number of covariates in each model, a separate model was conducted for each C-BARQ subscale (i.e., Trainability, Nonsocial Fear, and Excitability). Factors in each model included age [continuous variable], sex [categorical variable; Male and Female], and the C-BARQ subscale score [continuous variable] as well as two-way interactions between age and the subscale score and sex and the subscale score. For all GLMs, non-significant interactions were removed in a stepwise fashion, starting with the interaction with the largest *p*-value, until the final model only included significant interactions, or all interactions were removed. If an interaction was significant and one of the factors in the interaction was a categorical variable, a separate model was run for each level of the categorical variable. To compare model fit, AIC values were calculated for each full model with all interactions included and each final model with all non-significant interactions removed. These values were compared to AIC values for null models which had all predictors removed, with a difference in AIC values greater than 2 indicating improved model fit. Independent-samples *t*-tests were also conducted to determine if testing location affected scores on any of the dependent variables. Data were analyzed using the lme4 (Version 1.1–34) package in R (RStudio, Version 2023.09.0, Boston, MA, U.S.A.).

Q-Q plots for each full model were visually assessed to check the dispersion of residuals. Variance inflation factors (VIFs) were also calculated for the predictor variables in each model to check for multicollinearity. Assessment of Q-Q plots indicated the residuals for each full model were not over-dispersed. In addition, low VIFs (VIFs < 1.13) were observed between predictors in each model, indicating a lack of multicollinearity. Q-Q plots were created using the stats package (Version 4.3.1) and VIFs were calculated using the car package (Version 3.1-2) in R (RStudio, Version 2023.09.0, Boston, MA, U.S.A.).

To determine if performance on the DST was related to performance on the DRT, overall percent correct on the DST was correlated with the dependent measures from the DRT using Spearman’s rank-order correlations. Because these correlations were associated with specific hypotheses, we did not use Bonferroni corrections for any correlations (Armstrong, 2014). Correlations were analyzed using the Hmisc (Version 5.1-1) package in R (RStudio, Version 2023.09.0, Boston, MA, U.S.A).

To assess interrater reliability, a second independent coder scored 20% of the videos for both tasks. Intraclass correlation coefficients (ICC) were calculated for the dependent measures for the DRT, and Cohen’s Kappa was calculated for choice on the DST. The ICC were computed using two-way random-effects models based on single ratings and absolute agreement. Tests were conducted using the irr (Version 0.84.1) package in R (Rstudio, Version 2023.09.0, Boston, MA, U.S.A.). For the DST, interrater reliability was strong for percent correct during delay testing (ICC = 1). Reliability between scores was also strong for total number of correct reversal trials (ICC = 1), trial number of the first correct reversal (ICC = 1) and the difference score (ICC = 0.99) on the DRT.

## Results

### Delayed-search task

Of the 48 puppies tested, 5 were excluded due to changes in task procedures, 7 were excluded due to behavioral complications (e.g., stress, lack of motivation, distracted by owners), 10 failed to meet the warm-up criteria, and 1 failed the odor control. In addition, 2 dogs did not make a choice on one or more trials during the delay portion of the task and were removed from analyses. Therefore, 23 (13 F, 10 M; age: *M* = 7.04, *SE* = 0.48, range = 4–11) puppies were included in the analyses.

**Fig. 3:**
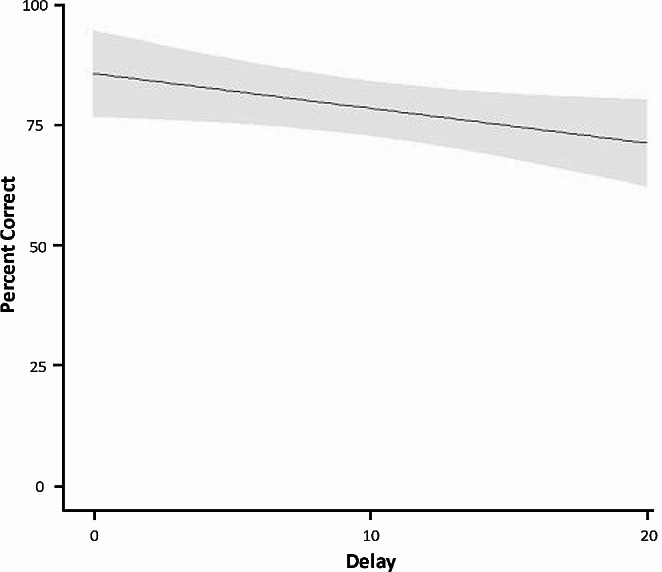
Percent correct as a function of increasing delay on the delayed-search task. Confidence bands represent 95% confidence intervals (CI)

There was a significant main effect of delay (LMER: *t*(45) = -2.49, *p* = .02) on percent correct, indicating that as delay increased, percent correct decreased, as depicted in Fig. 3. One-sample *t*-tests indicated that puppies performed significantly above chance (33%) at all delays, *t*(22)s > 7.4, *p*s < 0.001, indicating that puppies were proficient at the task at all delays despite a decrease in the percentage of correct trials at higher delays.

Full results for all GLMs for overall percent correct on the DST can be found in Table S3. For the trainability model for overall percent correct on the DST, addition of predictors did not improve fit relative to the null model (ΔAIC < 2), and all effects were not significant (*p*s > 0.06).

**Fig. 4:**
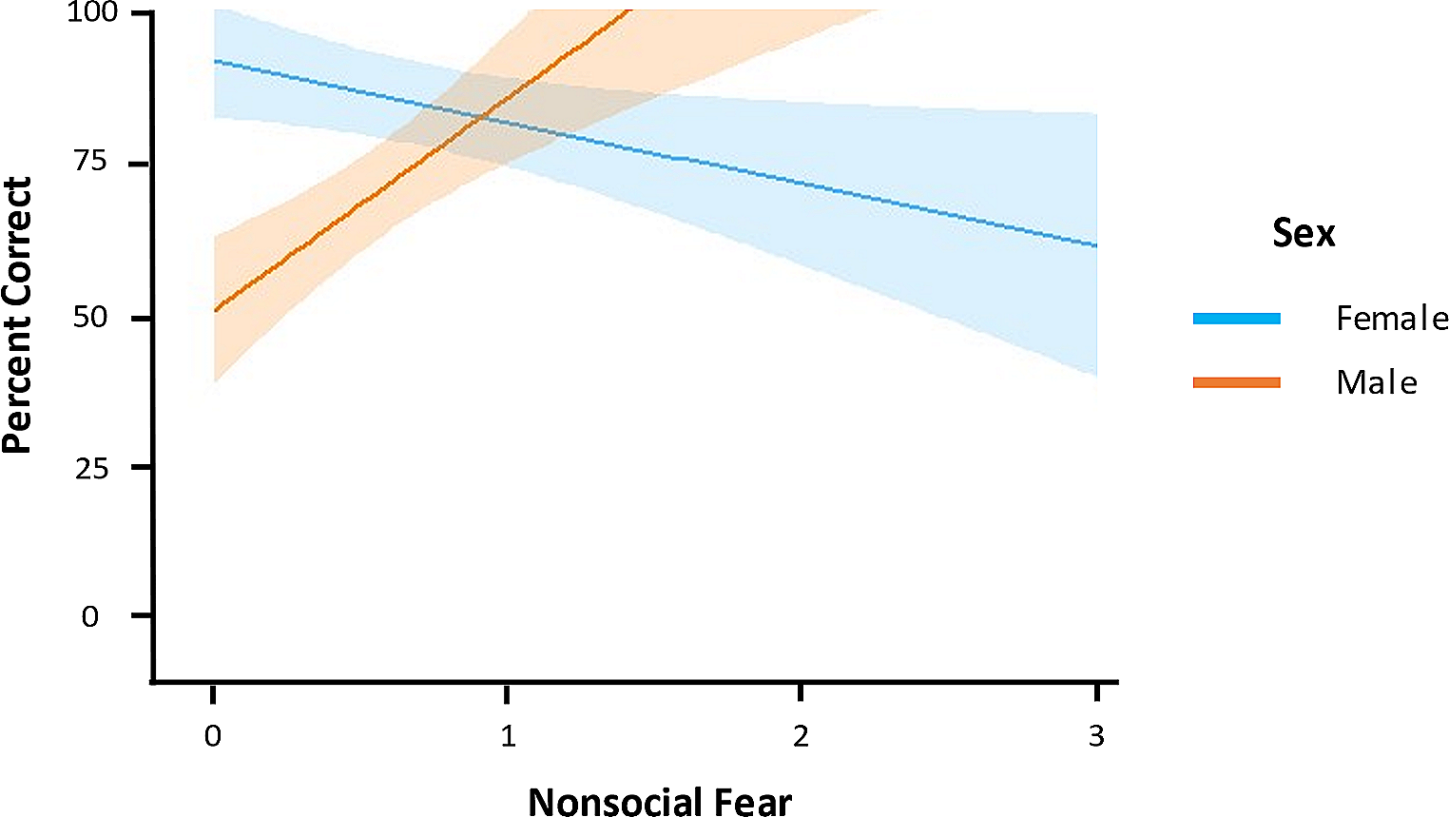
Interaction between sex and nonsocial fear on overall percent correct on the delayed-search task. The blue line indicates the function of nonsocial fear and percent correct for females (*n* = 13), and the orange line indicates the same function for males (*n* = 10). Confidence bands represent 95% CI

Addition of predictors did improve fit relative to the null model for both the full and final nonsocial fear models (ΔAIC > 2). A significant interaction was observed between sex and nonsocial fear (*p* < .001), so the effect of nonsocial fear on overall percent correct was analyzed separately for males and females. In females, nonsocial fear negatively predicted overall percent correct (GLM: *t* = -2.26, *p* = .047), whereas nonsocial fear positively predicted overall percent correct in males (GLM: *t* = 4.72, *p* = .002). Figure 4 illustrates the interaction between nonsocial fear and sex on overall percent correct on the DST. In the final nonsocial fear model, there were also significant main effects of sex (GLM: *t* = -5.63, *p* = < 0.001) and nonsocial fear (GLM: *t* = -2.37, *p* = .03); however, because both factors were involved in a significant interaction, conclusions were not drawn from these main effects. All other effects were not significant (*p*s > 0.23).

**Fig. 5:**
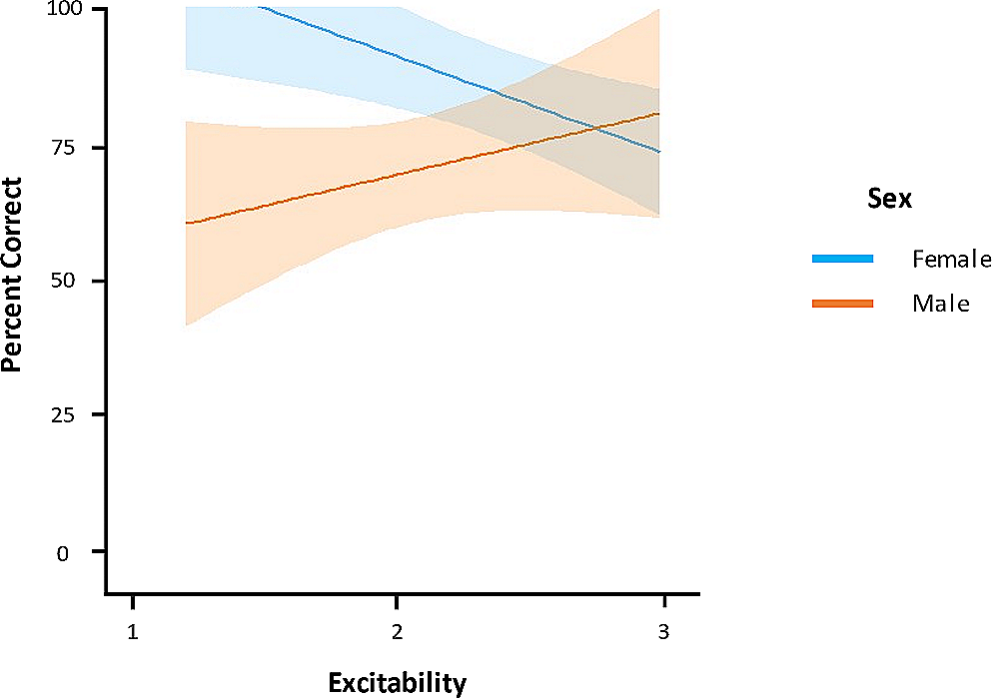
Interaction between sex and excitability on overall percent correct on the delayed-search task. The blue line indicates the function of excitability and percent correct for females (*n* = 13), and the orange line indicates the same function for males (*n* = 10). Confidence bands represent 95% CI

For the excitability model for overall percent correct, both the full and final models had improved fit relative to the null model (ΔAIC > 2). There was a significant interaction between sex and excitability (*p* = .01); therefore, the effect of excitability on overall percent correct was analyzed separately for males and females. In females, excitability negatively predicted overall percent correct (GLM: *t* = -7.08, *p* < .001), whereas no effect was observed for males (*p* = .33). Figure 5 depicts the interaction between sex and excitability on overall percent correct on the DST. There were also significant main effects of sex (GLM: *t* = -3.43, *p* = .003) and excitability (GLM: *t* = -2.99, *p* = .008) in the final excitability model; however, conclusions were not drawn from these main effects considering they were involved in a significant interaction. All other effects were not significant (*p*s > 0.14).

### Detour reversal task

For the DRT, 4 puppies were excluded due to behavioral issues that prevented completion of the task. An acquisition criteria was established after data collection and prior to data analyses to ensure that only puppies that demonstrated high accuracy in the acquisition phase of the task (i.e., 3 or more correct acquisition trials; see Brucks et al. 2017) and therefore experienced a true reversal during the reversal phase of the task were included in the final analyses. Of the puppies tested, 6 were unable to meet the acquisition criteria. An additional subset of puppies (*n* = 8) was tested with a starting location that was 2 m away from the barrier instead of 1 m due to experimenter error; however, distance from the barrier did not significantly affect any of the dependent measures for this task (*p* > .54), so these puppies were included in the final analyses. Overall, 38 (22 F, 16 M; Age: *M* = 6.0, *SE* = 0.4, range = 3–11) puppies were included in the final analyses. One puppy did not have a value for the difference score due to a malfunction with the video recording.

Full results for all GLMs for the total number of correct reversal trials and the first correct reversal trial number can be found in Tables S4 and S5. Addition of predictors led to worse fit for all models for both the total number of correct reversal trials and the first correct reversal trial (i.e., null models had lower AIC values, with ΔAIC > 2). In addition, no significant effects were observed in the final models for trainability (*p*s > 0.40), nonsocial fear (*p*s > 0.40), and excitability (*p*s > 0.48) for the total number of correct reversal trials and the first correct reversal trial number.

**Fig. 6:**
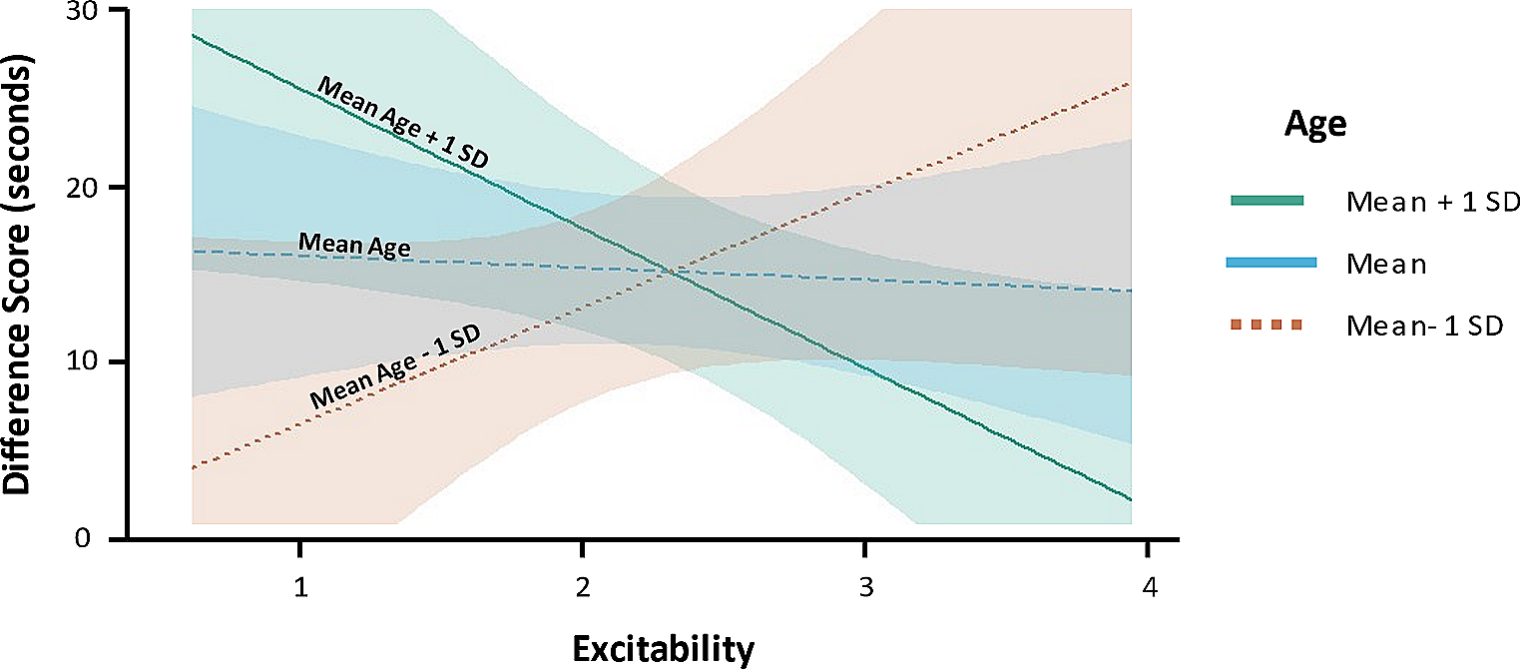
The difference score (i.e., latency on the first reversal trial – latency on the last acquisition trial) on the detour reversal task as a function of excitability for varying ages (orange = mean – 1 SD; blue = mean; green = mean + 1 SD). The lines illustrate predicted linear trajectories for each age with confidence bands representing 95% CI. A larger difference score indicates a greater increase in latency (i.e., the puppy took more time to navigate around the barrier) on the first reversal trial relative to the last acquisition trial

Full results for all GLMs for the difference score models can be found in Table S6. Trainability and nonsocial fear models had significantly reduced fit relative to the null model (lower AIC value for null model, with ΔAIC > 2). The excitability model demonstrated no difference in fit compared to the null model (ΔAIC < 2). However, a significant interaction was observed between age and excitability (GLM: *t* = -2.37, *p* = .02) in the final excitability model for the difference score. Figure 6 depicts the interaction between age and excitability on the difference score. Significant main effects of age (GLM: *t* = 2.44, *p* = .02) and excitability (GLM: *t* = 2.12, *p* = .04) were also observed in the final excitability model, but because these factors were involved in a significant interaction, conclusions were not drawn from these main effects. All other effects in the excitability model and the models for trainability and nonsocial fear were not significant (*p*s > 0.16).

In addition, testing location did not affect performance on the DST (*p* = .68) or the DRT (*p*s > 0.46).

### Between-task correlations

All correlations between overall percent correct on the DST and the dependent measures from the DRT were not statistically significant (*p*s > 0.14). Correlation values for all between-task correlations are listed in Table 1.

**Table 1:** Correlation values for the between-task correlations

Variable	Total Number of Correct Reversal Trials (DRT)	First Correct Reversal Trial Number (DRT)	Difference Score (DRT)
Overall Percent Correct (DST)	-0.38	0.38	0.27

## Discussion

Companion dogs under a year old were tested on the DST and DRT to corroborate previous findings demonstrating the ontogenetic development of cognition in working dogs and to evaluate other factors that can influence cognitive performance. Our results indicate that individual differences in temperament, age, and sex appear to influence cognitive performance in puppies. Contrary to our hypothesis, age did not positively predict performance on either task. However, interactions between sex and C-BARQ subscales indicate that differences in nonsocial fear and excitability influence performance during delay testing on the DST, but the effect varies between males and females. In addition, an interaction between excitability and age suggests that variations in excitability may differentially influence performance on the DRT depending on the age of the dog, with higher excitability leading to better performance in older dogs but worse performance in younger dogs. Lastly, no significant correlations were observed between the dependent measures of the two tasks despite both tasks purporting to measure aspects of EF. Although these results indicate that specific factors may influence cognitive performance in young companion dogs, the conclusions of the study are limited due to small sample sizes. However, this study provides a preliminary foundation for future research to continue to identify how individual differences in puppies may influence performance on cognitive tasks.

Despite previous studies demonstrating age-related improvements in cognitive performance in young puppies during the first years of development (Bray et al. 2021; Lazarowski et al. 2020b), age did not positively predict performance on either the DST or the DRT in this sample of puppies, although a range of ages across 3 to 11 months was tested. The high variability between individuals across many different factors (e.g., breed, place of acquisition, etc.) along with the relatively small sample sizes for both tasks likely contributed to a lack of an age effect. In addition, we used a cross-sectional design rather than a longitudinal design in this study, limiting our ability to track individual changes in performance across development. In previous studies, cognitive performance was evaluated in relatively homogenous and controlled populations, specifically a population of purpose-bred detection dogs (Lazarowski et al. 2020) and a population of dogs bred and raised for service work (Bray et al. 2021). These studies also had the opportunity to test many dogs across multiple timepoints. Therefore, age likely influences performance on these tasks in companion dogs as has been demonstrated in other populations, but we were unable to detect these effects due to the high variability in our sample and the reduced power associated with our experimental design and statistical analyses. This conclusion is additionally supported by our findings indicating that other factors outside of age (i.e., individual differences in temperament and sex) impacted cognitive performance in this sample.

Both nonsocial fear and excitability differentially affected overall percent correct on the DST across sexes. Specifically, nonsocial fear and excitability negatively predicted overall percent correct in females, indicating that as scores for both C-BARQ subscales increased, overall percent correct decreased in females. However, in contrast to our hypotheses, nonsocial fear in males positively predicted overall percent correct (i.e., as nonsocial fear increased, percent correct also increased), while excitability had no impact on performance on the DST. One potential explanation for these findings is that they illustrate how differences in baseline levels of arousal influence performance on this task across sexes. Both nonsocial fear (Souza et al. 2018) and excitability (Bray et al. 2015) appear to be related to emotional arousal in dogs, although they signify different valences of arousal (i.e., nonsocial fear is typically indicative of a negative valence of arousal, while excitability is related to a positive valence of arousal). In females, both forms of arousal appear to negatively impact performance on the DST, supporting findings in other studies indicating that higher levels of nonsocial fear (Overall et al. 2019) and excitability (Bray et al. 2017) led to worse performance on problem-solving and cognitive tasks. However, greater nonsocial fear improved males’ performance on the DST. Because anxious or stressful states have been shown to promote attentional selectivity towards potentially threatening stimuli (Fox et al. 2005), dogs experiencing moderate levels of fear may attend more heavily to the reward and test stimuli, increasing vigilance during delay testing. Although higher levels of nonsocial fear improved performance during delay testing in males, these puppies generally exhibited moderately low levels of nonsocial fear, and males had less variability in nonsocial fear (*M* = 0.56, *SE* = 0.15, range = 0–1.2) than females (*M* = 0.71, *SE* = 0.22, range = 0–3.2). Therefore, it is possible that this finding could be influenced by a lack of higher nonsocial fear scores in males. Additional research is needed to confirm if these relationships are consistent in a larger sample size with increased variability in temperament traits and to determine why variations in these traits between sexes may differentially influence performance on cognitive tasks.

On the DRT, an interaction between age and excitability revealed that excitability also influenced the speed in which the puppies navigated around the barrier on the first reversal trial, but this effect depended on the age of the puppy. Specifically, higher levels of excitability in younger puppies resulted in larger difference scores (i.e., the puppy took longer to navigate around the barrier on the first reversal trial relative to the last acquisition trial) whereas excitability in older puppies led to lower difference scores. This finding suggests that certain temperament traits may differentially affect cognitive performance within the first year of development. Although temperament is generally considered to be stable across development (Fratkin et al. 2013), the saliency of specific stimuli or environments may change as dogs age. In another study, Bray et al. (2015) found that dogs performed optimally on an inhibitory control task when they experienced median levels of arousal. Specifically, dogs with higher levels of baseline arousal (i.e., highly excitable dogs) demonstrated more inhibitory control in low-arousal contexts, whereas low excitability dogs performed better when arousal within the testing environment was elevated. Because more excitable dogs exhibit less inhibitory control in high arousal contexts, it is possible that a novel testing environment induces arousal in young puppies with high excitability, impairing performance on the DRT. However, older puppies would likely have more experience in different environmental contexts, potentially diminishing the level of arousal experienced by a novel testing environment. If the saliency of the testing environment was reduced in older puppies, this would potentially allow more excitable puppies to perform well on the DRT, whereas less excitable puppies would perform worse. Future research should seek to directly measure the level of arousal displayed by young companion dogs during cognitive testing, to determine if differences in arousal during testing exist across various testing environments, ages, and temperaments. In addition, it is important to note that the final model with this finding did not have improved fit relative to the null model. Therefore, caution should be taken when extracting conclusions from this finding, as they may be limited due to low sample sizes resulting in reduced statistical power.

Although effects of age, sex, and temperament were observed in our sample across both tasks, we observed no relationship in performance between tasks. This finding contrasts our hypothesis that performance across tasks would be related, which would have suggested that both tasks were measuring aspects of EF. Previous studies have used these tasks as measures of EF in dogs (Bray et al. 2021; Foraita et al. 2021b; Krichbaum et al. 2021; Lazarowski et al. 2020b; Osthaus et al. 2010); however, some studies also found no relationships between these two tasks (Lazarowski et al. 2020) or other tasks purported to measure similar aspects of EF (Foraita et al. 2021b), while others either only used one task (Krichbaum et al. 2021; Osthaus et al. 2010) or did not evaluate relationships between tasks (Bray et al. 2021). Several studies have even found that tasks expected to measure the same component of EF, namely inhibitory control, exhibit no relationships between each other (Bray et al. 2014; Brucks et al. 2017; Fagnani et al. 2016; Vernouillet et al., 2018), with other factors such as variations in task demands influencing performance across tasks. Detour tasks may also not be an appropriate measure of inhibitory control if differences in experience with the test stimuli or motivation for the reward are not accounted for across individuals (van Horik et al., 2018). More sensitive measures of detour performance may also be needed to accurately measure constructs such as flexibility or inhibitory control. Specifically, analyses of detour paths may be necessary to observe differences in performance between individuals and provide greater precision in detour behavior relative to correct or incorrect choices on specific trials. Ultimately, more research is needed to develop methodological controls that further confirm if these tasks are valid measures of EF and evaluate the impact of other factors (e.g., contextual or motivational differences) on task performance.

While the findings of this study provide insight into potential factors that may influence cognitive performance in dogs, there are several limitations that likely impacted the results of this research. Most notably, puppies tested on both tasks varied across many different factors, including breed, place of acquisition (i.e., breeder, stray, or rescue), and neuter status. In addition, the home environments of the companion dogs in our sample likely differed in many aspects, influencing the daily experiences and reinforcement histories of the dogs. Because of this variability, puppies may have had differing experience with the stimuli used in both tasks, and individuals may have had previous testing experience that was not recorded. Although it is important to test variable populations of dogs like the one sampled for this study to confirm findings observed in more homogenous populations, large sample sizes are needed to clearly observe potential effects. Therefore, the data presented in this study provide a preliminary foundation for future studies evaluating cognitive performance in young companion dogs, but ultimately, more data are needed to confirm the findings observed in this study and analyze how differences in these variables could ultimately impact cognitive performance.

A larger age range may also be needed to observe age-related improvements in cognitive abilities in companion dogs due to the wide variation between individuals. While Lazarowski et al. (2020) observed age effects between 3 and 12 months of age, Bray et al. (2021) tested dogs across 2 years of age; therefore, future studies should expand the range of ages tested to further examine these effects. In addition, other factors which could influence performance on cognitive tasks (e.g., trainability) may exhibit a non-linear trajectory during early and adolescent development (Asher et al. 2020). Although no effects of trainability were observed in this study, future studies should consider this factor and its subsequent impact on cognitive task performance.

In addition, while the C-BARQ has been validated for use in companion dogs, the initial validation study eliminated dogs that were less than a year old (Hsu and Serpell 2003). A more recent study in a working dog population validated the use of the survey at 6 months of age (Duffy and Serpell 2012), while another study observed relationships between C-BARQ subscales and performance on a behavioral evaluation in another working dog population as early as 3 months of age (Lazarowski et al. 2021). Therefore, additional research is needed to confirm the validity of this measure in a young companion dog population. Future studies should also compare C-BARQ subscale scores to behaviors exhibited during testing on cognitive tasks and in other experimental scenarios to determine whether temperament traits as characterized by the C-BARQ are consistent with behaviors observed during testing.

In sum, our findings provide a preliminary foundation regarding potential factors that appear to affect cognitive performance in a diverse sample of young companion dogs. While previous studies have used relatively homogenous populations of puppies to evaluate the ontogenetic development of cognition in dogs (Bray et al. 2021; Lazarowski et al. 2020b), this study is the first to use a companion dog population to measure the effect of age on cognitive task performance in puppies. The overall lack of age effects in our study is likely due to the diverse sample of dogs tested which led to other individual differences influencing cognitive task performance in addition to age. However, as more research reveals various factors affecting cognitive task performance in dogs, it will become pertinent for all studies evaluating canine cognition to consider the individual factors that may be influencing their conclusions.

## Electronic supplementary material

Below is the link to the electronic supplementary material.

Supplementary Material 1

## Data Availability

The data presented in this study are available on request from the corresponding author.
