# Supplementary material for: A preliminary analysis of the effect of individual differences on cognitive performance in young companion dogs
Source: Anim Cogn. 2024 Apr 1;27(1):30. doi: 10.1007/s10071-024-01868-4 (PMC10984887; doi:10.1007/s10071-024-01868-4)
Supplement: Supplementary file 1 — Supplementary Material 1 [file 10071_2024_1868_MOESM1_ESM.docx]

**Supplementary Information**

**Title:** A preliminary analysis of the effect of individual differences on cognitive performance in young companion dogs

**Journal:** Animal Cognition

**Authors:** Jordan G. Smith*, Sarah Krichbaum, Lane Montgomery, Emma Cox, and Jeffrey S. Katz

*Department of Psychological Sciences and Canine Performance Sciences, Auburn University, [jag0125@auburn.edu](mailto:jag0125@auburn.edu)

**Table S1. Demographic information of all dogs recruited for the study and those included in the analyses for each task.**

| **Dog Name** | **Age (Months)** | **Sex** | **Breed** | **Neuter Status** | **Acquisition** | **Completed DST** | **Completed DRT** |
| --- | --- | --- | --- | --- | --- | --- | --- |
| Annie | 6 | Female | Mixed Breed | Intact | Stray | ✓ | ✓ |
| Archer | 5 | Male | Labrador Retriever | Intact | Breeder | ✓ | ✓ |
| Artie | 5 | Female | Pit Bull Mix | Neutered | Rescue |  | ✓ |
| Asher | 6 | Male | Standard Poodle | Neutered | Breeder | ✓ |  |
| Bailey | 8 | Female | Great Pyrenees Newfoundland Mix | Intact | Breeder |  | ✓ |
| Bailey Lou | 7 | Female | Labrador Retriever | Intact | Breeder | ✓ | ✓ |
| Blanton | 5 | Male | Labrador Retriever | Intact | Breeder | ✓ | ✓ |
| Bonnie | 9 | Female | Pit Bull Mix | Neutered | N/A |  |  |
| Bradley | 7 | Male | Australian Cattle Mix | Intact | Stray |  | ✓ |
| Brady | 3 | Male | Golden Doodle | Intact | Breeder |  | ✓ |
| Chief | 4 | Male | Yorkshire Terrier | Intact | Breeder |  | ✓ |
| Clementine | 3 | Female | Golden Retriever | Intact | Breeder |  | ✓ |
| Davis | 9 | Male | Golden Retriever | Intact | Breeder |  | ✓ |
| Doc | 11 | Male | Labrador Retriever | Intact | Breeder | ✓ |  |
| Duke | 8 | Male | Pit Bull Mix | Neutered | Rescue |  | ✓ |
| Fred | 7 | Male | Pug | Intact | Rescue |  | ✓ |
| Georgia Peach | 5 | Female | Snazu | Neutered | Breeder |  | ✓ |
| Ginger | 3 | Female | Pit Bull Mix | Intact | Rescue |  | ✓ |
| Granger | 10 | Male | Labrador Mix | Neutered | Rescue | ✓ | ✓ |
| Harold | 5 | Male | Great Dane | Intact | Breeder |  |  |
| Ivy | 4 | Female | Catahoula Pit Bull Mix | Neutered | Rescue | ✓ |  |
| June | 7 | Female | Labrador Mix | Neutered | Rescue |  |  |
| K.O. | 6 | Male | Belgian Tervuren | Intact | Breeder | ✓ | ✓ |
| Kenzie | 8 | Female | Blue Heeler Mix | Neutered | Rescue | ✓ |  |
| Lucy | 4 | Female | Boykin Spaniel | Intact | Breeder | ✓ | ✓ |
| Mabel | 6 | Female | Aussiedoodle | Intact | Breeder | ✓ | ✓ |
| Maggie | 5 | Female | Pit Bull Mix | Neutered | Rescue | ✓ | ✓ |
| Mako | 9 | Female | Bloodhound | Intact | Breeder |  | ✓ |
| Mango | 11 | Female | Australian Labradoodle | Intact | Breeder | ✓ | ✓ |
| Mavis | 6 | Female | German Shepherd | Intact | Breeder |  | ✓ |
| Max | 4 | Male | Golden Doodle | Intact | Breeder |  | ✓ |
| Nellie | 6 | Female | Golden Doodle | Intact | Breeder | ✓ | ✓ |
| Odesza | 6 | Female | Australian Shepherd Mix | Intact | Rescue | ✓ | ✓ |
| Opal | 8 | Female | Labrador Mix | Intact | Rescue |  | ✓ |
| Oscar | 7 | Male | Scottish Terrier Mix | Neutered | Rescue | ✓ | ✓ |
| Pearl | 8 | Female | Labrador Mix | Intact | Rescue |  | ✓ |
| Pepper | 10 | Female | Great Pyrenees Newfoundland Mix | Neutered | Breeder |  |  |
| Percy | 10 | Male | Schnoodle | Neutered | Breeder | ✓ |  |
| Perrin | 4 | Male | Labrador Retriever | Intact | Breeder | ✓ | ✓ |
| Poppy | 10 | Female | Mixed Breed | Neutered | Rescue | ✓ | ✓ |
| Radar | 6 | Male | German Shepherd | Intact | Breeder |  | ✓ |
| Scout | 7 | Female | Great Pyrenees Mix | Neutered | Rescue | ✓ | ✓ |
| Stella | 3 | Female | Spitz Mix | Intact | Rescue |  | ✓ |
| Trigger | 10 | Male | Mixed Breed | Neutered | Rescue | ✓ |  |
| Tucker | 3 | Male | Australian Shepherd | Intact | Breeder |  | ✓ |
| Tucker | 3 | Male | Labrador Retriever | Intact | Breeder |  | ✓ |
| Winnie | 4 | Female | German Shepherd | Intact | Breeder |  | ✓ |
| Winnie | 8 | Female | Golden Retriever | Intact | Breeder | ✓ | ✓ |

**Table S2. Description of all items associated with the trainability, nonsocial fear, and excitability subscales from the Canine Behavioral Assessment and Research Questionnaire (C-BARQ)**

| **Subscale** | **Item Number** | **Item Description** |
| --- | --- | --- |
| Trainability | 1 | When off the leash, returns immediately when called. |
| Trainability | 2 | Obeys the "sit" command immediately |
| Trainability | 3 | Obeys the "stay" command immediately. |
| Trainability | 4 | Seems to attend/listen closely to everything you say or do. |
| Trainability | 5 | Slow to respond to correction or punishment; "thick-skinned". |
| Trainability | 6 | Slow to learn new tricks or tasks. |
| Trainability | 7 | Easily distracted by interesting sights, sounds, or smells. |
| Trainability | 8 | Will "fetch" or attempt to fetch sticks, balls, or objects. |
| Nonsocial Fear | 38 | In response to sudden or loud noises (e.g. vacuum cleaner, car backfire, road drills, objects being dropped, etc.). |
| Nonsocial Fear | 41 | In heavy traffic. |
| Nonsocial Fear | 42 | In response to strange or unfamiliar objects on or near the sidewalk (e.g. plastic trash bags, leaves, litter, flags flapping, etc.). |
| Nonsocial Fear | 44 | During thunderstorms, firework displays, or similar events. |
| Nonsocial Fear | 47 | When first exposed to unfamiliar situations (e.g. first car trip, first time in elevator, first visit to veterinarian, etc.). |
| Nonsocial Fear | 48 | In response to wind or wind-blown objects. |
| Excitability | 63 | When you or other members of the household come home after a brief absence. |
| Excitability | 64 | When playing with you or other members of your household. |
| Excitability | 65 | When doorbell rings. |
| Excitability | 66 | Just before being taken for a walk. |
| Excitability | 67 | Just before being taken on a car trip. |
| Excitability | 68 | When visitors arrive at your home. |

**Table S3. Model fit and results for overall percent correct on the delayed-search task (DST). Full model indicates model with all predictors and interactions while final model indicates model with non-significant interactions removed. Significant p-values (*p* < .05) are bolded.**

|  |  |  |  | **Coefficients** | | | |  | **Model Fit** | |
| --- | --- | --- | --- | --- | --- | --- | --- | --- | --- | --- |
| **Model** | **Distribution** | **Link** | **Predictor** | **ß** | ***SE*** | ***t*** | ***p*** |  | ***AIC* (Full Model)** | ***AIC* (Final Model)** |
| **Null Model** | Gaussian | Identity |  |  |  |  |  |  | 202.01 |  |
|  |  |  | Intercept | 78.76 | 3.82 | 20.62 | <0.001 |  |  |  |
|  |  |  |  |  |  |  |  |  |  |  |
| **Trainability Final Model** | Gaussian | Identity |  |  |  |  |  |  | 200.28 | 201.40 |
|  |  |  | Age | 1.44 | 1.63 | 0.88 | 0.39 |  |  |  |
|  |  |  | Sex | -14.39 | 7.26 | -1.98 | 0.06 |  |  |  |
|  |  |  | Trainability | 10.22 | 8.26 | 1.24 | 0.23 |  |  |  |
|  |  |  | Intercept | 47.68 | 23.27 | 2.05 | 0.06 |  |  |  |
| **Nonsocial Fear Final Model** | Gaussian | Identity |  |  |  |  |  |  | 185.94 | 185.17 |
|  |  |  | Age | 1.39 | 1.12 | 1.25 | 0.23 |  |  |  |
|  |  |  | Sex | -41.56 | 7.38 | -5.63 | **<0.001** |  |  |  |
|  |  |  | Nonsocial Fear | -10.27 | 4.33 | -2.37 | **0.03** |  |  |  |
|  |  |  | Sex*Nonsocial Fear | 45.83 | 9.17 | 5.00 | **<0.001** |  |  |  |
|  |  |  | Intercept | 82.43 | 8.70 | 9.47 | <0.001 |  |  |  |
|  |  |  |  |  |  |  |  |  |  |  |
| **Excitability Final Model** | Gaussian | Identity |  |  |  |  |  |  | 193.23 | 194.29 |
|  |  |  | Age | 1.75 | 1.43 | 1.23 | 0.23 |  |  |  |
|  |  |  | Sex | -79.81 | 23.27 | -3.43 | **0.003** |  |  |  |
|  |  |  | Excitability | -17.74 | 5.94 | -2.99 | **0.008** |  |  |  |
|  |  |  | Sex*Excitability | 29.15 | 10.43 | 2.80 | **0.01** |  |  |  |
|  |  |  | Intercept | 114.09 | 16.35 | 6.98 | <0.001 |  |  |  |

**Table S4. Model fit and results for total number of correct reversal trials on the detour reversal task (DRT). Full model indicates model with all predictors and interactions while final model indicates model with non-significant interactions removed. Significant p-values (*p* < .05) are bolded.**

|  |  |  |  | **Coefficients** | | | |  | **Model Fit** | |
| --- | --- | --- | --- | --- | --- | --- | --- | --- | --- | --- |
| **Model** | **Distribution** | **Link** | **Predictor** | **ß** | ***SE*** | ***z*** | ***p*** |  | ***AIC* (Full Model)** | ***AIC* (Final Model)** |
| **Null Model** | Poisson | Log |  |  |  |  |  |  | 114.96 |  |
|  |  |  | Intercept | 0.91 | 0.10 | 8.78 | <0.001 |  |  |  |
|  |  |  |  |  |  |  |  |  |  |  |
| **Trainability Final Model** | Poisson | Log |  |  |  |  |  |  | 123.18 | 120.35 |
|  |  |  | Age | 0.03 | 0.05 | 0.58 | 0.56 |  |  |  |
|  |  |  | Sex | -0.01 | 0.21 | -0.02 | 0.98 |  |  |  |
|  |  |  | Trainability | 0.07 | 0.19 | 0.38 | 0.70 |  |  |  |
|  |  |  | Intercept | 0.56 | 0.53 | 1.04 | 0.30 |  |  |  |
| **Nonsocial Fear Final Model** | Poisson | Log |  |  |  |  |  |  | 123.68 | 120.48 |
|  |  |  | Age | 0.03 | 0.05 | 0.68 | 0.5 |  |  |  |
|  |  |  | Sex | -0.01 | 0.21 | -0.04 | 0.97 |  |  |  |
|  |  |  | Nonsocial Fear | -0.01 | 0.16 | -0.08 | 0.93 |  |  |  |
|  |  |  | Intercept | 0.72 | 0.35 | 2.04 | 0.04 |  |  |  |
|  |  |  |  |  |  |  |  |  |  |  |
| **Excitability Final Model** | Poisson | Log |  |  |  |  |  |  | 123.15 | 119.99 |
|  |  |  | Age | 0.02 | 0.05 | 0.45 | 0.65 |  |  |  |
|  |  |  | Sex | -0.001 | 0.21 | -0.01 | 0.996 |  |  |  |
|  |  |  | Excitability | 0.10 | 0.14 | 0.71 | 0.48 |  |  |  |
|  |  |  | Intercept | 0.55 | 0.41 | 1.35 | 0.18 |  |  |  |

**Table S5. Model fit and results for first correct reversal trial number on the DRT. Full model indicates model with all predictors and interactions while final model indicates model with non-significant interactions removed. Significant p-values (*p* < .05) are bolded.**

|  |  |  |  | **Coefficients** | | | |  | **Model Fit** | |
| --- | --- | --- | --- | --- | --- | --- | --- | --- | --- | --- |
| **Model** | **Distribution** | **Link** | **Predictor** | **ß** | ***SE*** | ***z*** | ***p*** |  | ***AIC* (Full Model)** | ***AIC* (Final Model)** |
| **Null Model** | Poisson | Log |  |  |  |  |  |  | 114.33 |  |
|  |  |  | Intercept | 0.90 | 0.10 | 8.63 | <0.001 |  |  |  |
|  |  |  |  |  |  |  |  |  |  |  |
| **Trainability Final Model** | Poisson | Log |  |  |  |  |  |  | 122.68 | 119.65 |
|  |  |  | Age | -0.02 | 0.05 | -0.39 | 0.70 |  |  |  |
|  |  |  | Sex | 0.02 | 0.21 | 0.09 | 0.93 |  |  |  |
|  |  |  | Trainability | -0.11 | 0.19 | -0.61 | 0.54 |  |  |  |
|  |  |  | Intercept | 1.28 | 0.52 | 2.48 | 0.01 |  |  |  |
| **Nonsocial Fear Final Model** | Poisson | Log |  |  |  |  |  |  | 123.05 | 119.97 |
|  |  |  | Age | -0.03 | 0.05 | -0.53 | 0.59 |  |  |  |
|  |  |  | Sex | 0.03 | 0.22 | 0.14 | 0.89 |  |  |  |
|  |  |  | Nonsocial Fear | 0.04 | 0.17 | 0.22 | 0.82 |  |  |  |
|  |  |  | Intercept | 1.01 | 0.35 | 2.88 | 0.004 |  |  |  |
|  |  |  |  |  |  |  |  |  |  |  |
| **Excitability Final Model** | Poisson | Log |  |  |  |  |  |  | 123.38 | 119.98 |
|  |  |  | Age | -0.02 | 0.05 | -0.45 | 0.66 |  |  |  |
|  |  |  | Sex | 0.02 | 0.21 | 0.09 | 0.93 |  |  |  |
|  |  |  | Excitability | -0.03 | 0.14 | -0.21 | 0.83 |  |  |  |
|  |  |  | Intercept | 1.09 | 0.40 | 2.70 | 0.007 |  |  |  |

**Table S6. Model fit and results for difference score (first reversal trial latency (s) – last acquisition trial latency (s)) on the DRT. Full model indicates model with all predictors and interactions while final model indicates model with non-significant interactions removed. Significant p-values (*p* < .05) are bolded.**

|  |  |  |  | **Coefficients** | | | |  | **Model Fit** | |
| --- | --- | --- | --- | --- | --- | --- | --- | --- | --- | --- |
| **Model** | **Distribution** | **Link** | **Predictor** | **ß** | ***SE*** | ***t*** | ***p*** |  | ***AIC* (Full Model)** | ***AIC* (Final Model)** |
| **Null Model** | Gaussian | Identity |  |  |  |  |  |  | 277.55 |  |
|  |  |  | Intercept | 13.05 | 1.63 | 8.03 | <0.001 |  |  |  |
|  |  |  |  |  |  |  |  |  |  |  |
| **Trainability Final Model** | Gaussian | Identity |  |  |  |  |  |  | 284.41 | 282.78 |
|  |  |  | Age | 0.23 | 0.81 | 0.29 | 0.78 |  |  |  |
|  |  |  | Sex | -2.41 | 3.44 | -0.70 | 0.49 |  |  |  |
|  |  |  | Trainability | 0.53 | 3.00 | 0.18 | 0.86 |  |  |  |
|  |  |  | Intercept | 11.28 | 8.43 | 1.34 | 0.19 |  |  |  |
| **Nonsocial Fear Final Model** | Gaussian | Identity |  |  |  |  |  |  | 284.28 | 282.82 |
|  |  |  | Age | 0.26 | 0.79 | 0.33 | 0.74 |  |  |  |
|  |  |  | Sex | -2.44 | 3.48 | -0.70 | 0.49 |  |  |  |
|  |  |  | Nonsocial Fear | -0.06 | 2.67 | -0.02 | 0.98 |  |  |  |
|  |  |  | Intercept | 12.47 | 5.79 | 2.16 | 0.04 |  |  |  |
|  |  |  |  |  |  |  |  |  |  |  |
| **Excitability Final Model** | Gaussian | Identity |  |  |  |  |  |  | 278.17 | 278.24 |
|  |  |  | Age | 7.76 | 3.18 | 2.44 | **0.02** |  |  |  |
|  |  |  | Sex | -0.46 | 3.33 | -0.14 | 0.89 |  |  |  |
|  |  |  | Excitability | 19.91 | 9.39 | 2.12 | **0.04** |  |  |  |
|  |  |  | Age*Excitability | -3.37 | 1.43 | -2.37 | **0.02** |  |  |  |
|  |  |  | Intercept | -30.84 | 20.33 | -1.52 | 0.14 |  |  |  |
